# Translational control of *furina* by an RNA regulon is important for left-right patterning, heart morphogenesis and cardiac valve function

**DOI:** 10.1242/dev.201657

**Published:** 2023-11-30

**Authors:** Agnieszka Nagorska, Andreas Zaucker, Finnlay Lambert, Angus Inman, Sara Toral-Perez, Jan Gorodkin, Yue Wan, Michael Smutny, Karuna Sampath

**Affiliations:** ^1^ Warwick Medical School, University of Warwick, Gibbet Hill Road, Coventry CV4 7AL, UK; ^2^ Stem Cell and Regenerative Biology, Genome Institute of Singapore, A*STAR, Singapore 138672; ^3^ Center for non-coding RNAs in Technology and Health, Department of Veterinary and Animal Sciences, Faculty for Health and Medical Sciences, University of Copenhagen, Grønnega °rdsvej 3, 1870 Frederiksberg C, Denmark; ^4^ Centre for Mechanochemical Cell Biology, University of Warwick, Gibbet Hill Road, Coventry CV4 7AL, UK; ^5^ Centre for Early Life, University of Warwick, Gibbet Hill Road, Coventry CV4 7AL, UK

**Keywords:** Left-right asymmetry, FurinA, RNA element, Ybx1, 3′UTR, Translational control, CRISPR/Cas editing, Nodal signalling, Heart development, Cardiac morphogenesis, Valve regurgitation

## Abstract

Heart development is a complex process that requires asymmetric positioning of the heart, cardiac growth and valve morphogenesis. The mechanisms controlling heart morphogenesis and valve formation are not fully understood. The pro-convertase FurinA functions in heart development across vertebrates. How FurinA activity is regulated during heart development is unknown. Through computational analysis of the zebrafish transcriptome, we identified an RNA motif in a variant FurinA transcript harbouring a long 3′ untranslated region (3′UTR). The alternative 3′UTR *furina* isoform is expressed prior to organ positioning. Somatic deletions in the *furina* 3′UTR lead to embryonic left-right patterning defects. Reporter localisation and RNA-binding assays show that the *furina* 3′UTR forms complexes with the conserved RNA-binding translational repressor, Ybx1. Conditional *ybx1* mutant embryos show premature and increased Furin reporter expression, abnormal cardiac morphogenesis and looping defects. Mutant *ybx1* hearts have an expanded atrioventricular canal, abnormal sino-atrial valves and retrograde blood flow from the ventricle to the atrium. This is similar to observations in humans with heart valve regurgitation. Thus, the *furina* 3′UTR element/Ybx1 regulon is important for translational repression of FurinA and regulation of heart development.

## Teaser

**Summary:** An RNA element in the *furina* 3′ untranslated region recognises the RNA-binding protein Ybx1 and functions in translational control of FurinA in zebrafish embryos.

## INTRODUCTION

Cardiovascular disease is a leading cause of death and accounts for a quarter of all deaths in the UK (Cheema et al., 2022). Even though cardiovascular development has been extensively studied, the molecular mechanisms underlying congenital heart disease are not fully known. The importance of RNA-binding proteins (RBPs) and RNA motifs in regulating organ development and diseases has been recently uncovered and understanding their interactions is fundamental to developing future diagnostic and therapeutic strategies (Brinegar and Cooper, 2016; Kalsotra et al., 2008; Sephton et al., 2010).

Zebrafish is a popular research model for studying heart development because the embryos are transparent, allowing in-depth observation of formation of many internal organs using live microscopy until larval stages. Additionally, although the heart in zebrafish embryos develops by 24 h post-fertilisation (hpf), embryos can survive the first week of life without a circulatory system. Zebrafish heart development is a dynamic process: at 24 hpf, a primitive beating organ is already formed, and by 48 hpf looping of the chambers and atrioventricular (AV) canal formation is complete (Liu and Stainier, 2012). Although heart formation occurs rapidly, many complex processes must be completed for the functional organ to develop. One key process is the establishment of left-right (LR) asymmetry, which is required for the correct placement of internal organs, including the heart (Bakkers, 2011).

In mammals, positioning of the heart on the left side and liver on the right is referred to as *situs solitus*. Disruptions to this process may cause left or right isomerism disorders that affect ∼1:10,000 individuals (Blum and Ott, 2018). Following heart looping, morphogenesis of the valves is initiated in the AV canal by formation of the endocardial cushions, which gradually differentiate and form functional heart valves (Gunawan et al., 2019). At similar stages, zebrafish embryos are small enough to obtain the required oxygen to live by passive diffusion. Therefore, unlike other model organisms, many mutations in the zebrafish heart do not cause embryonic lethality within the first week of zebrafish life (Liu and Stainier, 2012). This is especially useful for studying key processes in cardiac differentiation, and cardiac valve and septal development, which if disrupted in mammals result in foetal death.

The mechanisms that control LR asymmetry are well-conserved in vertebrates (Hamada et al., 2002). For instance, asymmetric left-sided expression of the secreted growth factor Nodal during somitogenesis in the lateral plate mesoderm (LPM) has been reported in many organisms including frogs, fish, chicks and mice (Levin et al., 1995; Long et al., 2003, 1996; Sampath et al., 1997, 1998; Shiratori and Hamada, 2006; Varlet and Robertson, 1997). Motile cilia generate calcium transients in the LR organiser, and drive leftward *Nodal* gene expression (Francescatto et al., 2010; Shinohara and Hamada, 2017). Expression of the Nodal inhibitor Lefty1 in the embryonic midline spatially restricts Nodal to the left LPM and results in asymmetric expression of the Nodal target Pitx2 (Chen and Shen, 2004; Lin et al., 1999; Lowe et al., 1996; Schottenfeld et al., 2007; Shiratori and Hamada, 2006; Thisse and Thisse, 1999).

The pro-protein convertase FurinA cleaves Nodal and is essential for heart development in many vertebrates (Beck et al., 2002; Constam and Robertson, 2000). Studies in zebrafish showed that FurinA is essential for maturation of the Nodal homologue Southpaw (Spaw) and establishes its signalling range in the LPM. Mutations disrupting zebrafish FurinA result in heart looping and trabeculation defects. The *furina* mutant embryos also display abnormal jaw development and 80% of maternal zygotic mutants die owing to lack of inflation of the swim bladder (Tessadori et al., 2015; Walker et al., 2006; Zhou et al., 2021). In the mouse, deletions in Furin cause embryonic lethality, and the mutants display a range of cardiac morphogenesis defects, including ventral closure, abnormalities of outflow tract and severe heart looping defects. Furin mutant mice also exhibit impairment of large vessel formation and defects in yolk sac vasculature (Dupays et al., 2019; Roebroek et al., 1998). Conditional deletion of *Furin* in endothelial cells in mouse did not reduce the severity of phenotype, and heart valve malformations in this allele also resulted in embryonic lethality (Kim et al., 2012), underscoring the importance of Furin during cardiac development.

Although the molecular mechanisms underlying transcriptional control of the Nodal pathway have been extensively studied, very few studies have explored regulation of the LR axis by non-coding regions and post-transcriptional regulation (Minegishi et al., 2021).We found that many Nodal pathway components in zebrafish harbour a short, conserved 3′ untranslated region (3′UTR) motif characterised by a AGCAC sequence motif, followed by a predicted stem and loop structure motif. The 3′UTR element was first identified in *squint* (*sqt*) [(*nodal-related 1* (*ndr1*)] transcripts. A fluorescent *sqt* RNA reporter localises to dorsal progenitors in early zebrafish embryos (Gilligan et al., 2011; Gore et al., 2005; Kumari et al., 2013). We demonstrated that transcripts of the Nodal inhibitors *lefty1* and *lefty2* also contain the 3′UTR motif, and *lefty* reporter RNAs also localise in early embryos, indicating similar activities of the 3′UTR motifs (Zaucker et al., 2017). The conserved RBP Ybx1, binds the 3′UTR element in *sqt*, *lefty1* and *lefty2* and translationally represses these RNAs during early development (Kumari et al., 2013; Zaucker et al., 2017). Overexpression of full-length *lefty1/2-gfp* versus *lefty1/2* lacking the 3′UTR motif showed that deletion of the element leads to a strong loss-of-nodal phenotype through inhibition of Nodal (Zaucker et al., 2017). Mutations in mammalian *Ybx1* also led to de-repression of Nodal and Lefty in cultured cells, suggesting broader conservation of this mechanism.

Given that Ybx1 was found to translationally repress a range of different mRNAs through the 3′UTR motif, the motif was named ‘Ybx1 binding element’ (YBE) (Zaucker et al., 2019). By computational analysis of the zebrafish transcriptome, we now find that the YBE motif is present in a variant 3′UTR transcript (variant X1) of the Nodal maturation enzyme FurinA, but not in *spaw* (also known as *ndr3*). We show that the *furina* variant X1 harbours the YBE motif in its 3′UTR and is expressed prior to organ positioning in zebrafish. Disruptions of the *furina* 3′UTR element lead to LR patterning defects. Conditional *ybx1* mutant embryos exhibit LR axis defects and abnormal visceral organ positioning. In addition, these *ybx1* mutant embryos have an enlarged AV canal and show retrograde blood flow. This is similar to mitral valve regurgitation disorder, a condition in humans characterised by a leaky heart valve and back-flow of blood into the heart chambers. Our work shows that translational control by Ybx1 functions in LR axis formation, heart morphogenesis and valve development, and identifies a new zebrafish model for mitral valve regurgitation disorders.

## RESULTS

### Two *furina* transcript isoforms are expressed throughout embryonic development

**Fig. 1.**
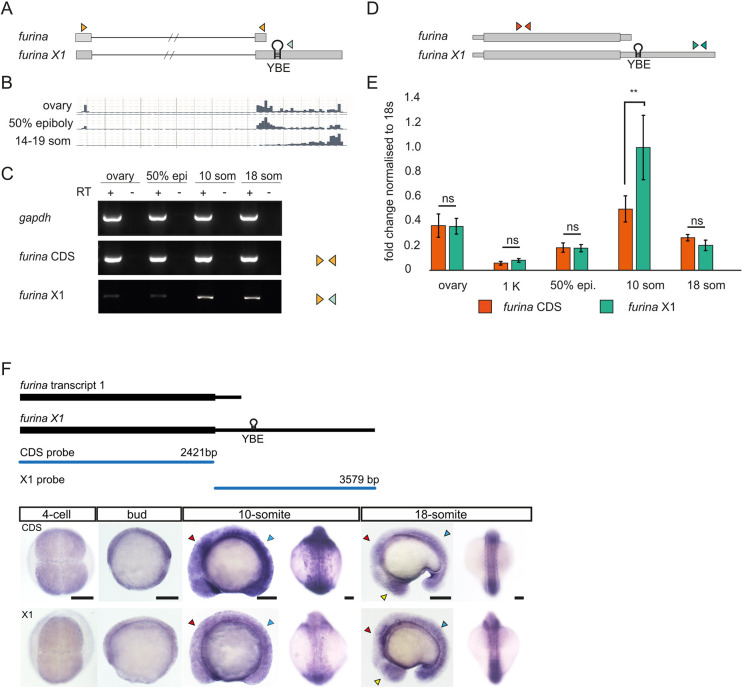
**Zebrafish *furina* transcripts show different 3′UTR lengths, with a predominant long isoform expressed during somitogenesis.** (A) Schematic of *furina* transcript 1 and variant X1. Orange arrowheads represent primers to amplify transcript 1; one orange and one green primer were used to detect variant X1. Hairpin indicates the YBE motif in the 3′UTR of X1. (B) RNA sequencing excerpt from ovary, 50% epiboly and 14-19 som stages. (C) Semi-quantitative RT-PCR detecting *gapdh* positive control, *furina* coding sequences (CDS) and *furina* variant X1 3′UTR (X1). (D) Schematic of primers used in qPCR to detect the *furina* CDS and variant X1. The orange primer set was used to amplify the CDS and green primers used to amplify variant X1. (E) Quantitative PCR analysis from ovary, 1 K, 50% epiboly, 10-som and 18-som embryos. Orange bars represent *furina* coding region, green bars show exclusively variant X1. **P<0.01 in unpaired, two-tailed Student's *t*-test. *n*=50 embryos per stage for mRNA extraction, *P*=0.014. ns, not significant. (F) Whole-mount *in situ* hybridisation with probes that detect the CDS or variant X1. Yellow arrowheads point to enrichment in eyes, red in hindbrain and blue in somites. Lateral and dorsal views are shown at 10-som and 18-som. Scale bars: 100 µm.

Previous studies have demonstrated that FurinA proprotein convertase cleaves Nodal and facilitates establishment of LR asymmetry (Constam and Robertson, 2000; Tessadori et al., 2015). Through analysis of zebrafish genomic databases, we found that, in addition to the previously described *furina* transcript, a long *furina* variant X1 is also expressed during zebrafish embryogenesis. Variant X1 consists of an exceptionally large (∼4 kb) 3′UTR sequence that has been only computationally predicted. Analysis of available RNA-sequencing datasets shows that during somitogenesis, the *furina* variant X1 is expressed at levels higher than the previously identified short *furina* isoform (White et al., 2017; Fig. 1A,B). To determine whether variant X1 can be detected in embryos, we collected cDNA libraries from adult fish ovaries and 50% epiboly, 10-somite (10-som) and 18-somite (18-som) developmental stages and performed semi-quantitative PCR. In agreement with the RNA-sequencing data, we observed that the predicted variant X1 was not efficiently amplified in the ovary and at 50% epiboly. The highest expression levels of the variant were detected during somitogenesis (Fig. 1C). To quantify the relative expression levels of the transcript isoforms, we performed quantitative real-time PCR (qRT-PCR) with distinct primers to detect the coding sequences (CDSs) or variant X1 3′UTR, and normalised to 18S rRNA control (Fig. 1D,E). We did not detect a significant difference between the expression levels of the two transcripts in the ovary, at the 1 K cell stage or at 50% epiboly. Transcript X1 levels were high at 10-som and the isoforms were detected at similar levels by 18-som (Fig. 1E). Next, we examined whether there are differences in the expression pattern of the variant isoform in zebrafish embryos. Consistent with previous work (Tessadori et al., 2015), a *furina* coding region probe detected ubiquitous expression at all stages examined: 4-cell stage, bud, 10-som and 18-som. The expression pattern of variant X1 was similar to the Refseq-annotated transcript and no obvious differences were observed in lateral or dorsal views, although whole-mount *in situ* hybridisation (WISH) to detect the CDS gave a slightly stronger signal during early somitogenesis (Fig. 1F). At 10-som and 18-som, we noticed that there were areas where both transcripts were slightly enriched. Overall, we did not find any obvious difference in the spatial distribution of the two isoforms (Fig. 1F). Thus, both *furina* transcripts are ubiquitously expressed, throughout embryonic development, with a slight enrichment in the eyes, hindbrain, and somite structures.

### The *furina* X1 variant harbours the YBE element

**Fig. 2.**
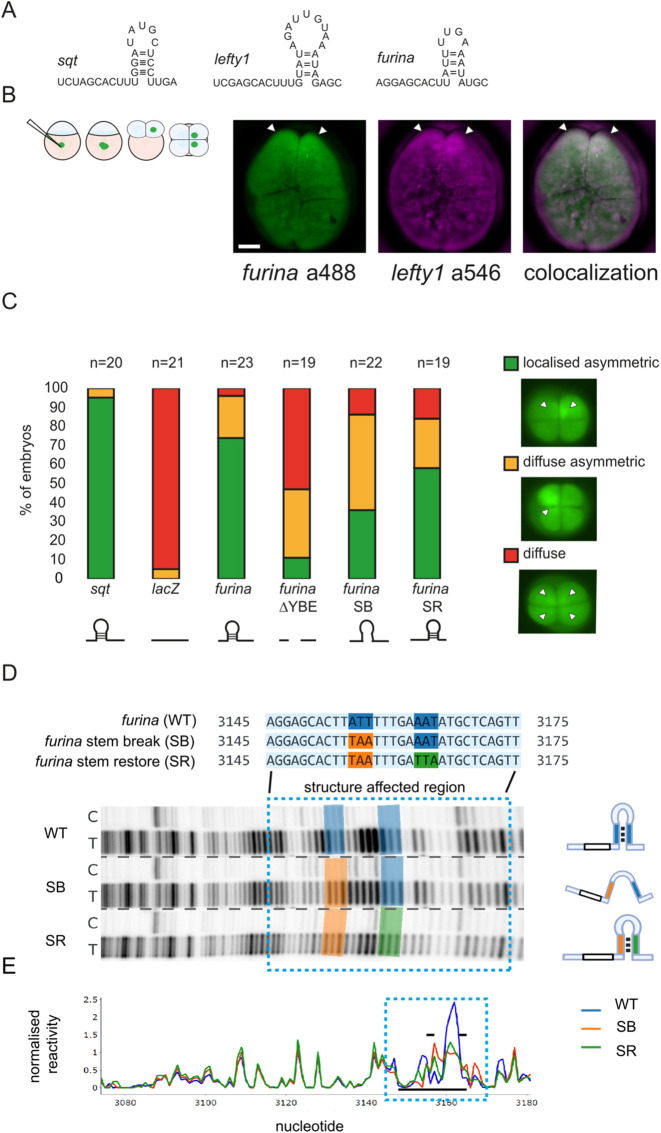
**The *furina* variant X1 transcript harbours a YBE motif in the 3′UTR.** (A) schematic showing hairpin YBE motifs in *sqt*, *lefty1* and *furina* variant X1. (B) Left: schematic of localisation assay. Embryos were injected into the yolk at the 1-cell stage and mRNA localisation was analysed at the 4-cell stage. Right: confocal images showing colocalisation of *furina* (labelled with Alexa 488-UTP) fluorescent reporter and *lefty1* (labelled with Alexa 546-UTP) in zebrafish embryos at the 4-cell stage. *n*=11. Arrowheads indicate fluorescent reporter mRNA localisation. Scale bar: 20 µm. (C) Stacked bar graph showing localisation categories in fluorescent reporter mRNA-injected embryos: localised asymmetric (green), diffuse asymmetric (yellow) and diffuse (red). Schematic representations of fluorescent RNAs of control *sqt* (*n*=20), *lacZ* (*n*=21) and *furina* variant X1 RNAs (full-length furina; *n*=23), or disrupting the YBE motif (ΔYBE; *n*=19; SB, *n*=22; SR, *n*=19) are shown below in black. (D) Structure probing of *furina* X1 3′UTR. Structural mutations were generated based on the predicted stem loop (SB, SR). Structure footprint gels for *furina* RNAs. Blue highlighted sequences (top) and blue dotted box (bottom) indicates the structure affected region. Lanes labelled C indicate control untreated RNA that was reverse transcribed; lanes labelled T indicate RNA treated with NAI-N3 before reverse transcription. Dashed lines separate cropped regions from different parts of the same gel. Schematics on the right represent the predicted YBE structure in WT, SB and SR. (E) Quantification of structure footprint gels. Blue dotted box represents the structure affected region (blue highlighted sequences). Black bars indicate the predicted YBE stem loop structure.

We previously found that many Nodal pathway components harbour a 3′UTR element containing an AGCAC sequence motif, followed by a predicted stem and a variable length loop region (hairpin) (Zaucker et al., 2017). Computational analysis identified the *furina* variant X1 as a potential mRNA with a YBE element in the 3′UTR (Fig. 2A). The YBE-harbouring *sqt* RNA localises to one or two cells at the 4-cell stage (Gore et al., 2005; Gilligian et al., 2011) and the YBE in *lefty1* and *lefty2* mRNAs can localise exogenous reporter RNAs similar to *sqt* (Zaucker et al., 2017). If the *furina* YBE motif behaves similarly to the 3′UTR motif in these RNAs, they should colocalise in early zebrafish embryos. To test this hypothesis, *furina* variant X1 and *lefty1* fluorescent mRNA reporters were co-injected into 1-cell-stage embryos and analysed for colocalisation at the 4-cell stage. The *furina* variant X1 fluorescent mRNA reporter colocalised with *lefty1* in all injected embryos (*n*=16; Fig. 2B). To determine whether mutations affecting the 3′UTR YBE motif disrupt reporter mRNA localisation at the 4-cell stage, we generated deletion constructs that are predicted to change the YBE structure. The fluorescent reporter *furina* ΔYBE had the entire YBE motif deleted within the 3′UTR. *furina* Stem-break (SB) had a mutation disrupting base pairing within the stem of the YBE, and *furina* Stem-restore (SR) had a swapped sequence that restores base pairing within the stem of the hairpin. Injected fluorescent *lacZ*-βglobin mRNA served as a negative control and *sqt* RNA as a positive control. Embryos were injected with a fluorescent reporter and then scored for reporter RNA localisation at the 4-cell stage. *lacZ*-βglobin fluorescent mRNA reporter distribution was diffuse everywhere in the blastoderm, but, in contrast, *sqt* RNA localised asymmetrically in one or two cells in 95% of embryos (*n*=19) (Fig. 2C). *furina* variant X1 fluorescent reporter localised asymmetrically in 75% of embryos (*n*=17), whereas deletion of the YBE motif resulted in mislocalisation of the mRNA in 90% of embryos (*n*=18). Breaking of the stem caused 15% of SB reporter-injected embryos to show complete mislocalisation and 45% showed diffuse asymmetric distribution of the reporter. Injection with SR restored the localisation by 25% compared with SB (Fig. 2C). Thus, the *furina* X1 variant harbours a functional YBE motif.

The *furina X1* UTR was also investigated by *in vitro* RNA structure probing (Fig. 2D,E). In the wild-type *furina* (*furina* WT), we observed a region of increased probe reactivity, flanked by regions of relatively low probe reactivity. Reactivity with the probe indicates high probability of single-strandedness, and this pattern approximately corresponded to the predicted stem-loop region in Fig. 2A. The region (blue dashed boxes) in the footprinting gel shows that there is a change in the pattern of bands from the wild-type pattern in the region encompassing the YBE region in the *furina* SB mutant, and this pattern is partially restored to the wild-type pattern in the *furina* SR mutant. The pattern noticeably flattened in *furina* SB, but was partially restored in *furina* SR and appeared more similar to the wild-type pattern (Fig. 2D). Quantification of the gel footprints (Fig. 2E) showed that the RNA structure outside of the YBE was largely unchanged in all RNAs tested, suggesting that structural mutations within the YBE only cause local changes in structure when probed *in vitro*. Comparing the quantified pattern of RNA structure by Pearson correlation (0.62 for WT versus SB and 0.84 for WT versus SR) indicated partial loss of the overall RNA structure in SB and partial restoration in SR. This partial loss and restoration of structure aligns with the *in vivo* reporter assays in Fig. 2C. Therefore, the *furina* X1 variant harbours a functional YBE motif with a structured RNA motif.

### Ybx1 pull-down reveals interactions with the *furina* variant X1

**Fig. 3.**
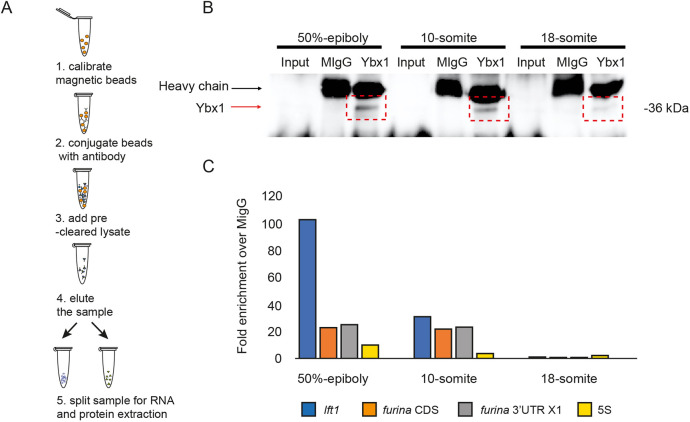
**Ybx1 protein interacts with *furina* variant X1 mRNA.** (A) Schematic of RNA immunoprecipitation of embryo lysates with anti-Ybx1 antibodies. (B) Representative western blot showing pull-down of Ybx1 from 50% epiboly, 10-som and 18-som embryonic lysates. *n*=250 embryos per stage; black arrow indicates IgG heavy chain; red arrow indicates Ybx1 band. (C) qPCR analysis of samples from immunoprecipitation. Fold enrichment normalised to input and mIgG control are shown for *lefty1* (blue), *furina* coding region (orange), *furina* variant X1 3′UTR (grey) and 5S negative control (yellow).

The RBP Ybx1 binds to the YBE motif in the 3′UTR of Nodal and Lefty mRNAs and forms a YBE/Ybx1 translational repression and RNA localisation module (Kumari et al., 2013; Zaucker et al., 2017). We hypothesised that the YBE we identified in the *furina* X1 variant RNA is likely to bind to Ybx1. To determine whether Ybx1 interacts with *furina* X1 mRNA *in vivo*, we performed RNA immunoprecipitation with zebrafish embryonic lysates from various stages (50% epiboly, 10-som and 18-som) using anti-Ybx1 antibodies, followed by qRT-PCR (Fig. 3). In western blots, we detected Ybx1 protein at 50% epiboly, 10-som and, to a lesser extent, at 18-som. Pull-downs with mouse IgG served as a negative control (Fig. 3B). Consistent with our published work, *lefty1* was enriched at 50% epiboly and the 10-som stage. Using primers to distinguish between the *furina* coding sequence versus 3′UTR sequences, we found that at 50% epiboly and 10-som, there is enrichment of the *furina* long isoform over mouse IgG controls in pull-downs in both set of samples, whereas no significant difference in *furina* isoforms was detected in pull-downs from 18-som embryo lysates (Fig. 3C). Ribosomal 5S amplification served as a negative control in all cases, and *lefty1* served as positive control. These results indicate that *furina* mRNA is in a complex with Ybx1 protein at 50% epiboly and at 10-som, but not at 18-som.

### Variant X1 of *furina* is translationally repressed by Ybx1 in zebrafish embryos

**Fig. 4.**
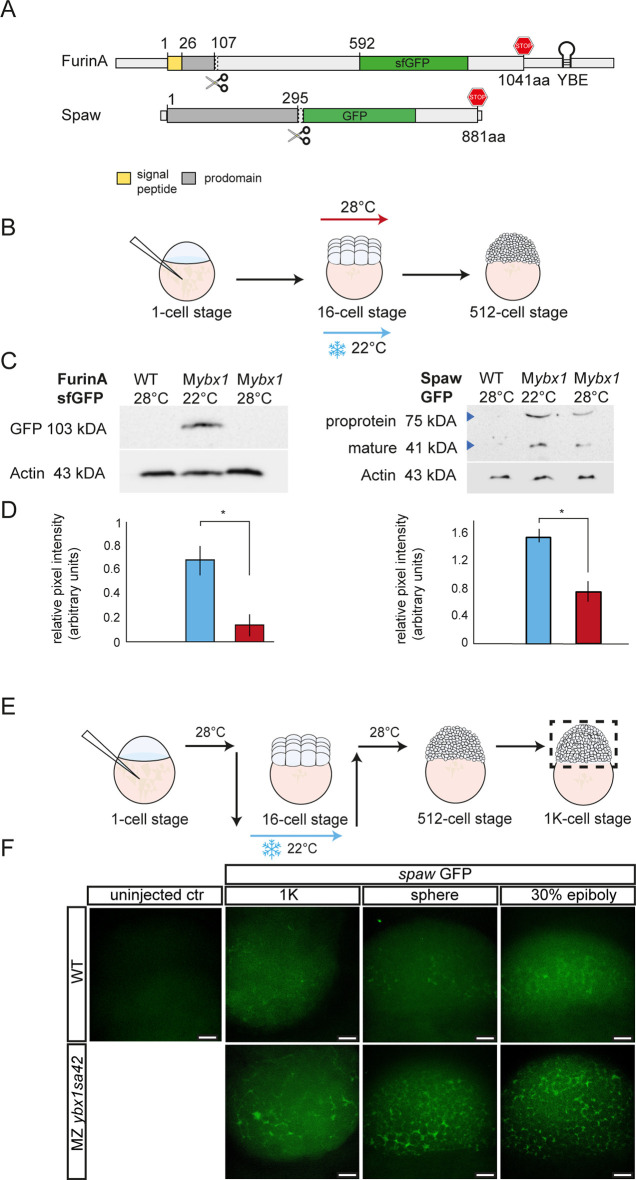
**FurinA translation and Spaw maturation is increased in *ybx1* mutant embryos.** (A) Schematic of GFP fusion reporters for *furina* X1 and *spaw*. Hairpin indicates the YBE motif in *furina* RNA. The FurinA pro-domain is shown in dark grey and the signal peptide in yellow; for Spaw, the pro-domain is indicated in dark grey; stop codon indicated in red in FurinA and Spaw. (B) Schematic of temperature shift of *ybx1^sa42^* mutant embryos. Embryos were injected with GFP reporter-fusion mRNA, grown at 28°C until the 16-cell stage, shifted to 22°C until the 512-cell stage, when protein was extracted. (C) Western blots of embryonic lysates showing FurinA-sfGFP (left) and Spaw-GFP fusion protein (right), following injection of embryos with mRNA reporter. Lanes were loaded with lysates from un-injected wild-type control, M*ybx1* at 22°C and M*ybx1* control embryos at 28°C. Actin loading control is aligned to the corresponding samples. (D) Bar graphs show quantification of corresponding band intensities, with FurinA-sfGFP (left) and mature Spaw (right) normalised to the Actin loading control, *P<0.05 (*P*=0.021 for FurinA-GFP; *P=*0.026 for Spaw-GFP)*.* (E) Schematic showing temperature shift of *ybx1* mutant embryos following injection with *spaw* GFP reporter. (F) Confocal images of 1 K, sphere and 30% epiboly wild-type (top) and *ybx1* mutant (bottom) embryos. WT, wild type. Scale bars: 50 µm.

The YBE in *nodal* and *lefty1/2* functions in translational control of these RNAs during early development. To test whether the *furina* variant X1 is similarly regulated by Ybx1, we performed our established translation assay that utilises *ybx1^sa42^* temperature-sensitive mutant fish to measure the expression of microinjected reporter constructs in *ybx1* mutants by circumventing the early embryonic death phenotype of *ybx1* null mutants (Kumari et al., 2013). The Ybx1^sa42^ mutant protein is expressed in embryos at the permissive temperature, but Ybx1^sa42^ protein levels are reduced in embryos at the restrictive temperature (Kumari, 2013). Cold-shock treatment during cleavage and blastula stages leads to developmental arrest during gastrulation and the embryonic phenotypes at 22°C are similar to *ybx1* null mutants (Kumari et al., 2013). Therefore, to examine exogenous Furin reporter protein expression, we injected *ybx1* mutant embryos at the 1-cell stage with *furina-*sfGFP:*furina3′UTR* RNA. Ybx1 protein function was disabled between 16-cell and 512-cell stages, as shown in the schematic, and embryos were analysed for protein expression between 1 K and epiboly stages (Fig. 4A,B). Control wild-type embryos that underwent the same cold-shock treatment or maternal *ybx1* (M*ybx1*) mutant embryos kept at the permissive temperature of 28°C were used for comparison with mutant embryos. Analysis of the *furina*-GFP reporter showed a strong band in the M*ybx1* lane, but not in control embryo or uninjected control lanes. We quantified the expression levels based on band intensity and normalised to Actin levels. Significantly higher FurinA GFP levels were detected in lysates from *ybx1* mutant embryos (Fig. 4C,D).

As FurinA cleaves Spaw/Nodal to control the Nodal signalling range in the lateral plate mesoderm (Tessadori et al., 2015), we posited that overexpression of FurinA in *ybx1* mutant embryos would lead to increased processing of Spaw and aberrant Nodal signalling in *ybx1* mutant embryos. To test this, we injected *spaw*-GFP fusion mRNA into *ybx1* mutants at the 1-cell stage, followed by temperature shift at the 16-cell stage, and collected embryonic lysates at the 500-cell stage (Fig. 4A,B). We detected two Spaw protein products, consistent with the existence of a Spaw precursor (75 kDa) and a mature Spaw (41 kDa) (Fig. 4C). Interestingly, in mutant lysates, we found elevated levels of Spaw mature peptide compared with controls. Quantification of band intensity of Spaw mature domain (*n*=3 experiments) and normalisation to Actin loading control (Fig. 4D) showed that, in the absence of Ybx1 function, FurinA protein levels are upregulated. Therefore, Ybx1 translationally represses *furina* variant X1 mRNA.

Mature Nodal/Southpaw is secreted into the extracellular space. We therefore examined Spaw maturation in *ybx1* mutant embryos over time (Fig. 4E,F). We injected *spaw* GFP reporter at the 1-cell stage and analysed expression at the 1 K, sphere and 30% epiboly. At the 1 K, sphere and 30% epiboly stages, both intra- and extracellular GFP expression was detected by imaging the blastoderm of wild-type embryos (Fig. 4E,F). By contrast, in *ybx1* mutant embryos, there was an obvious increase in accumulation of Spaw-GFP in the extracellular space (Fig. 4F). We measured total Spaw-GFP fluorescence levels across the blastoderm in wild-type and *ybx1* mutant embryos and found that overall fluorescence levels were similar (Fig. S1). Therefore, even though overall Spaw-GFP levels were apparently similar in wild-type and mutant embryonic blastoderms, Spaw maturation by Furin is increased and faster in *ybx1* mutants, resulting in excess and premature secreted mature Nodal protein in mutant embryos.

### Mutant *ybx1^sa42^* embryos exhibit LR asymmetry defects

**Fig. 5.**
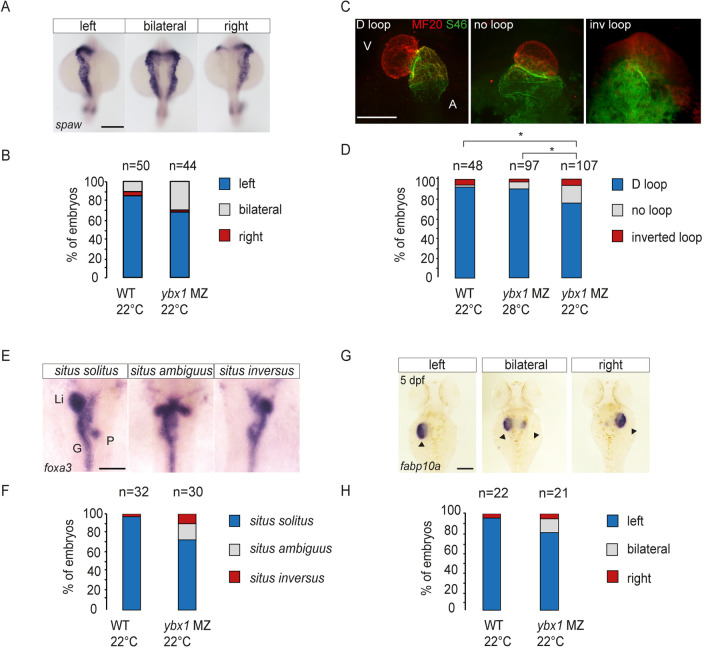
**Mutant *ybx1^sa42^* embryos have abnormal LR expression.** (A) WISH to detect *spaw* in wild-type (*n*=50) and *ybx1^sa42^* mutant (*n*=44) embryos at the 21-som stage. Scale bar: 100 µm. (B) Bar graph showing the proportion of embryos with *spaw* expression on the left (blue), bilaterally (grey) or on the right (red). Fisher's Exact two-tailed probability test *P*=0.0125. (C) Immunofluorescence showing MF20 labelling in the ventricle (red) and S46 labelling in the atrium (green) at 55 hpf. Representative D loop, no loop and inverted (inv) loop hearts are shown. A, atrium; V, ventricle. Scale bar: 100 µm. (D) Quantification of looping of the heart. Directional loop (D loop, blue), no loop (grey) and inverted looping (red) of the heart, with *ybx1* mutant embryos at 22°C (*n*=107) showing a higher percentage of inverted and no loop hearts compared with control wild-type (WT; *n*=48) or *ybx1* embryos at 28°C (*n*=97). An unpaired, two-tailed Student's t-test was used to analyse the data. *P<0.05. MZ*ybx1* 22°C compared with 28°C: *P*=0.022; MZ*ybx1* 22°C compared with WT: *P*=0.030. (E) WISH to detect *foxa3* at 55 hpf in wild-type and *ybx1* mutant embryos. G, gut; Li, liver; *P*, pancreas. Scale bar: 100 µm. (F) Quantification of visceral organ positioning in wild-type (*n*=32) and *ybx1* mutant (*n*=30) embryos at 55 hpf showing an increase in *situs ambiguus* (grey) and *situs inversus* (red) with a decrease in *situs solitus* (blue) in *ybx1* mutant embryos. Fisher's Exact two-tailed test. *P*=0.0113. (G) Expression of *fabp10a* at 5 dpf*.* Arrowheads indicate the liver. Scale bar: 100 µm. (H) Quantification of liver positioning in wild-type (*n*=22) and *ybx1* mutant (*n*=21) embryos showing *situs solitus* (blue), *situs ambiguus* (grey) and *situs inversus* (red).

Increased processing of Spaw/Nodal and altered Nodal levels are associated with LR asymmetry defects (Long et al., 2003; Tessadori et al., 2015). Therefore, we next investigated whether *ybx1* mutant zebrafish embryos show defects in LR asymmetry. WISH at 18-som showed a 15% increase in bilateral or right-sided *spaw* expression in *ybx1* mutants (Fig. 5A,B, *n*=44). Abnormal *spaw* expression is associated with organ laterality defects, such as defective looping of the heart and mispositioning of the liver, pancreas and gut (Long et al., 2003). In *ybx1* mutant embryos, we detected a 20% increase in an aberrant looping of the heart compared with both wild-type embryos at 22°C, and 28°C *ybx1* controls (Fig. 5C,D). A proportion of the temperature-shifted *ybx1* mutant embryos also showed no looping, or complete inversion of the heart chambers (Fig. 5C,D). Analysis of heart looping in *ybx1* mutant embryos showed altered heart morphology in mutant embryos compared with wild-type controls. Further examination of *bmp4* and *notch1b* expression in the heart showed that, in addition to the looping defects, *ybx1* mutants also have a larger heart (*n*=8/24) and AV canal (*n*=4/29), respectively (Fig. S2).

To assess the positioning of visceral organs, such as liver, gut and pancreas, we examined expression of *foxa3*. In *ybx1* mutants, we observed an increase in either duplication, mispositioning or inversion of the liver and pancreas (Fig. 5E,F). By contrast, the majority of control embryos showed correct organ positioning (Fig. 5E,F). Similarly, *fabp10a* expression revealed a similar trend: the majority of control embryos had a liver on the left-hand side and 20% of the *ybx1* mutant embryos had either duplicated livers or inverted liver position (Fig. 5G,H, black arrowheads). Therefore, LR organ positioning is affected in *ybx1* mutant embryos.

### Cardiac morphogenesis is affected in *ybx1^sa42^* mutant embryos

**Fig. 6.**
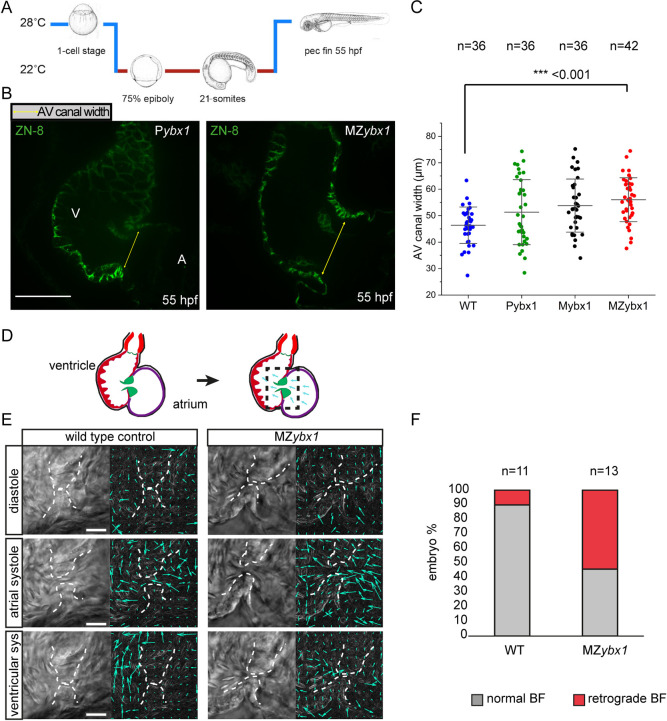
***Ybx1* mutant embryos show abnormal heart morphogenesis.** (A) Schematic of temperature shift experiments. *ybx1* mutant embryos and controls were shifted to 22°C at 75% epiboly until 21-som and then grown at 28°C until 55 hpf. (B) Confocal images of zebrafish heart showing immunofluorescence with the ZN-8 antibody to label the of atrioventricular (AV) canal. Yellow arrows indicate the width of the AV canal in control P*ybx1* and MZ*ybx1* mutant embryos. A, atrium; V, ventricle. Scale bar: 25 µm. (C) Quantification AV canal width in µm from immunostaining of wild-type (WT; blue, *n*=36), P*ybx1* (green, *n*=36), M*ybx1* (black, *n*=36) and MZ*ybx1* embryos (red, *n*=42). ANOVA test was used for statistical analysis (*P*=0.00016361; ***P<0.001). (D) Schematic of zebrafish heart at 5 dpf (left) and schematic of PIV analysis (right); black dashed box shows the area of the heart imaged and analysed. (E) Left: DIC images from movies of wild-type and *ybx1* mutant hearts during diastole, atrial systole and ventricular systole. Right: PIV analysis of the images; cyan arrows indicate movements of red blood cells. Scale bars: 20 µm. (F) Percentage of embryos showing normal blood flow (BF) versus retrograde BF in wild-type (*n*=11) and *ybx1* mutant (*n*=12) embryo groups.

To determine whether heart morphogenesis is altered in the *ybx1* mutant embryos, we performed immunofluorescence with the ZN8 antibody, which labels the AV canal, and quantified the AV canal width. At 55 hpf, we observed an overall increase in width of the AV canal compared with wild-type embryos. Additionally, measurements in wild-type controls were more consistent, whereas *ybx1* mutant embryos presented a broad range in AV canal width (Fig. 6A-C). This raised the possibility that *ybx1* mutant embryos harbour defects in heart valves and cardiac blood flow.

To determine the consequences of the larger AV canal in the mutants, we analysed the heartbeat of wild-type and MZ*ybx1* mutant embryos by live imaging at 5 days post-fertilisation (dpf) (Fig. 6D,E). The movement of red blood cells was tracked, and the direction of blood flow during the contraction of the heart muscle was assessed. Live imaging at different stages of the heartbeat cycle and tracking of the red blood cells at 5 dpf revealed that in seven out of 13 MZ*ybx1* mutant embryos red blood cells travelled back to the atrium, in contrast to wild-type controls, in which blood flowed from the atrium to the ventricle (Fig. 6E,F, Movies 1, 2). Quantification showed that over 50% of MZ*ybx1* mutants exhibited retrograde blood flow (Fig. 6F), which is consistent with abnormal heart morphogenesis in these embryos.

### Mutant *ybx1* embryos manifest heart valve regurgitation and reversed blood flow

**Fig. 7.**
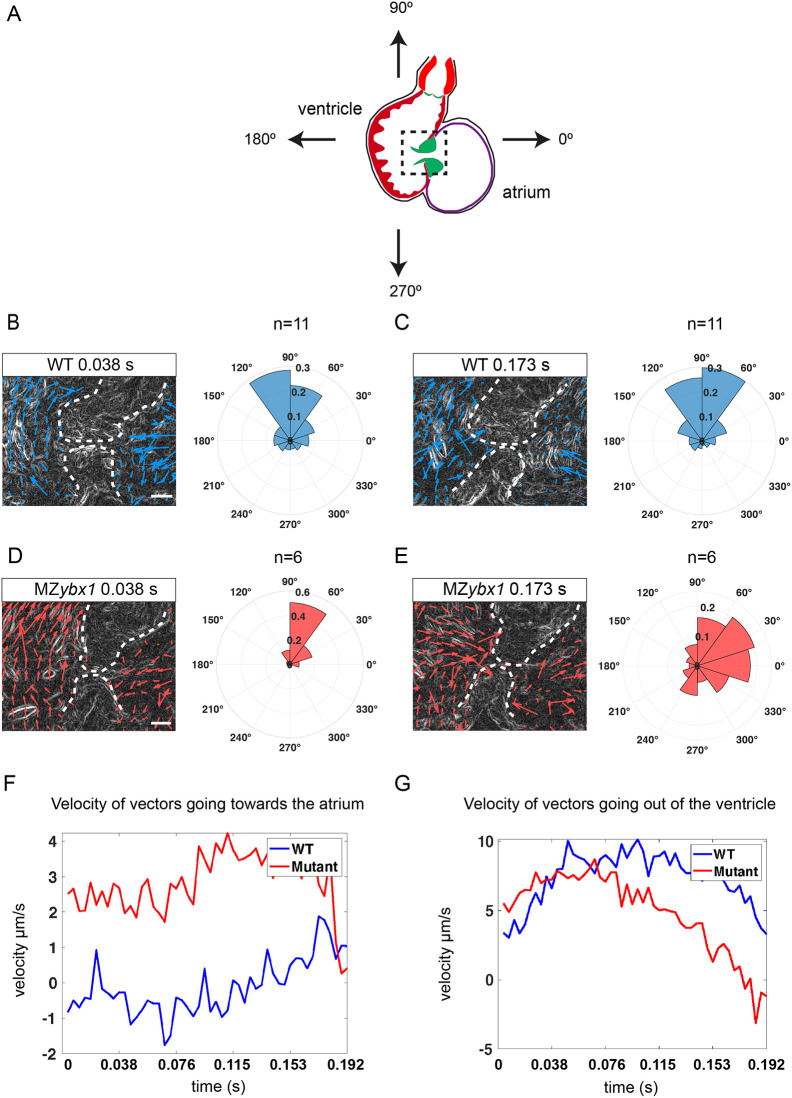
**Mutant *ybx1* embryos have retrograde blood flow at 5** **dpf.** (A) Schematic of a zebrafish heart at 5 dpf. Arrows indicate the directions of flow; black box shows the region analysed in PIV. (B) Representative image of a wild-type (WT) embryo, showing PIV application at 0.038 s of ventricular systole. Vectors generated from red blood cells are shown as blue arrows. Rose plot shows quantification of direction of the blood flow at 0.038 s for the imaged wild-type hearts (*n*=11). Scale bar: 25 µm. (C) A PIV image of wild-type ventricular contraction at 0.173 s (left) and quantification of direction of generated vectors (rose plot on right) (*n*=11). Vectors are shown as blue arrows. Scale bar: 25 µm (bars in B,D apply to C,E, respectively). (D) A PIV image showing the direction of blood flow in MZ*ybx1* embryos with retrograde blood flow at 0.038 s. Vectors are shown as red arrows. Rose plot shows analysis of direction of blood flow in MZ*ybx1* embryos with retrograde blood flow (*n*=6). Scale bar: 25 µm. (E) PIV analysis at 0.173 s of ventricular systole and quantification of vector direction in *ybx1* embryos with retrograde blood flow at 0.173 s (*n*=6). Vectors are shown as red arrows. Scale bar: 25 µm. Dashed lines in B-E delineate heart valves. (F) Analysis of average velocity of horizontal vectors moving towards the atrium over the duration of ventricular contraction. Wild-type embryos are shown in blue and *ybx1* mutants in red. (G) Analysis of average velocity of vertical vectors moving out of the ventricle during ventricular contraction. Wild-type embryos in blue and *ybx1* mutants in red.

Analysis of the *ybx1* heartbeat revealed that during the ventricular systole, instead of blood cells being pushed out of the ventricle, a large number of red blood cells leak through the heart valves and move back into the atrium. To examine blood flow patterns in these embryos in depth, we analysed and quantified the movements of the red blood cells around the AV canal (Fig. 7A). We focused on two timepoints during ventricular contraction in *ybx1* embryos with reverse blood flow (RF) versus wild-type controls. At the beginning of the ventricular systole, red blood cells moved in the same direction in wild-type and mutant embryos (Fig. 7B,D). However, at the end of the ventricular systole, in mutant embryos we observed that red blood cells change direction and start moving back, whereas in wild-type embryos the movement trajectory remained the same (Fig. 7C,E). Analysis of the velocity of the red blood cells during contraction of the ventricle confirmed that in *ybx1* RF embryos there is increase in cell movement back into the atrium, whereas in control embryos little movement was detected in this direction (Fig. 7F). Furthermore, in *ybx1* embryos, initially the cells moved out of the ventricle, but over time their velocity gradually decreased. In control embryos, however, movement of cells out of the ventricle remained steady and consistent (Fig. 7G). The altered movement of blood cells is consistent with our observations that *ybx1* mutants have a larger AV canal (Fig. 6B,C), and heart valves appeared misaligned, resulting in reverse blood flow, showing a heart defect at 5 dpf. This shows the important role of translational control by Ybx1 protein, not only in LR asymmetry establishment during early embryogenesis in zebrafish, but also in correct heart morphogenesis and heart valve function.

### Disruption of the *furina* X1 3′UTR leads to LR asymmetry defects

To test directly the role of the YBE region in the *furina* X1 3′UTR, we performed CRISPR-Cas9 genome editing using guide RNAs targeting the YBE region of *furina*. Despite several attempts, we were unable to recover stable germline mutants in which the *furina* X1 3′UTR YBE region had been disrupted, whereas stable germline *furina* ATG region mutants similar to those described by Tessadori et al. (2015) were recovered (Nagorska, 2022). Therefore, we generated somatic *furina* X1 mutants (i.e. ‘crispants’; Burger et al., 2016) using Cas9 protein and a specific guide RNA targeting the *furina* ATG or a pair of gRNAs flanking the *furina* 3′UTR YBE region.

**Fig. 8.**
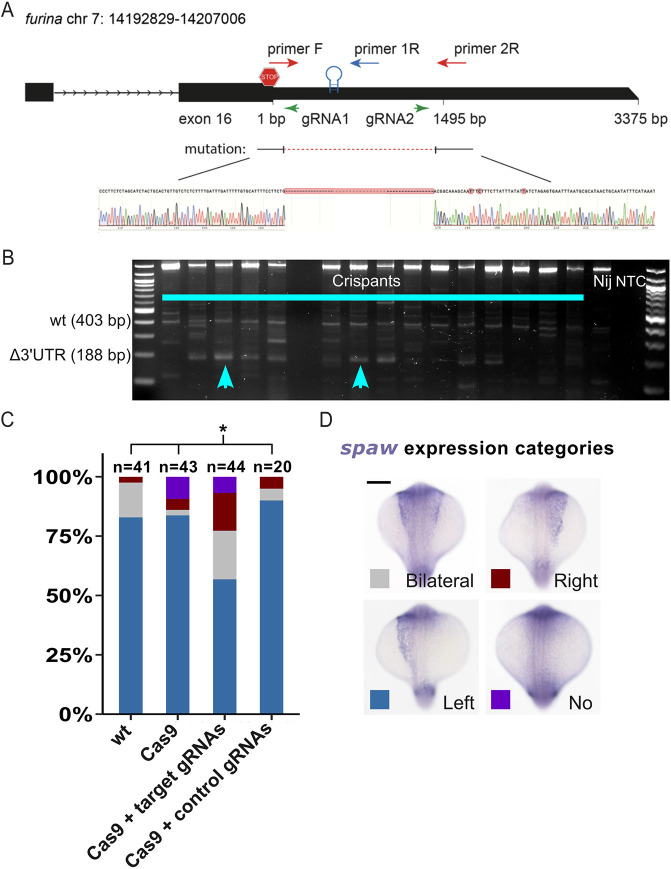
**Deletion of the *furina* 3′UTR YBE leads to abnormal LR patterning.** (A) Schematic of the *furina* X1 3′UTR genomic region on chromosome 7. Exon 16 contains the 3′ end of the CDS with the stop codon (stop sign) and the entire 3375 bp of the 3′UTR. The red dotted line shows the sequences deleted in *furina* Δ3′UTR crispants by the two gRNAs (green arrows). The position of primers used for the simultaneous detection of 3′UTR deletion alleles (primer F and primer 2R) with wild-type alleles (primer F and primer 1R) in three primer genotyping PCRs are indicated. (B) Gel image showing *furina* Δ3′UTR crispant genotyping PCRs using genomic DNA from 1 dpf embryos. The expected sizes of the wild-type (wt) and the Δ3′UTR deletion mutant PCR products are shown next to the 100 bp DNA ladder (far-left lane). Mutant bands (cyan arrowheads) together with wild-type bands are only observed with the crispant samples. The non-injected control (Nij) only shows the wild-type band, and no product is detected in the no template control lane (NTC). (C) Stacked bar chart showing the percentage of embryos falling into different categories for *spaw* expression by WISH on 19-21 som embryos: non-injected wild-type embryos, Cas9 protein only-injected embryos, Cas9 RNPs loaded with the two targeting gRNAs (gRNAs 1+2), and control Cas9 RNPs loaded with gRNA2 and a non-cutting control gRNA (gRNAc). *n*=number of embryos. *P<0.05 by Fisher's exact test. (D) Images of *spaw* expression by WISH in 19-21 som embryos showing the various categories shown in the bar graph in C.

The morphology of these embryos was found indistinguishable from controls at prim5 (Fig. 8). WISH to detect *spaw* in the lateral plate mesoderm showed that, in contrast to control embryos that typically have left-sided expression of *spaw* (83%, *n*=41), a significant proportion of *furina Δ3′UTR YBE* embryos showed disruptions in LR asymmetry with bilateral or right-sided *spaw* (∼43%, *n*= 44; Fig. 8). By contrast, control embryos injected with Cas9 protein alone or Cas9 together with a control gRNA showed LR patterns similar to those of wild-type embryos. These results support a role for the *furina* 3′UTR and YBE in regulation of asymmetric *spaw* expression and LR patterning.

## DISCUSSION

In this work, we describe a novel *furina* variant X1 mRNA with a YBE element in the 3′UTR. Similar to other *nodal* mRNAs, we show that *furina* YBE motif is recognised by the RBP Ybx1 and has an important role in translational repression of FurinA. Previous work by other groups showed that FurinA protein cleaves Spaw and establishes its signalling range (Tessadori et al., 2015). We observed that, in the absence of Ybx1, FurinA is premature and overexpressed and, as a result, Spaw processing is increased. This causes excess Spaw protein levels and ectopic Spaw expression outside the left lateral plate mesoderm. This leads to LR asymmetry defects, with abnormal heart looping and abnormal positioning of liver and pancreas. Our work thus identifies a previously unappreciated mechanism of regulation of LR asymmetry and cardiac morphogenesis through the RBP Ybx1. Although many transcription factors have been found to control LR patterning across animal species, very few RBPs and/or corresponding RNA elements have been identified in this process (Rago et al., 2019; Vejnar et al., 2019); therefore, our work could lead to potentially new clinical diagnostic tests for LR asymmetry defects that are currently unassigned.

RNA elements have a crucial regulatory role in mRNA biogenesis, stability, localisation and translation. The Hu RBP family in human neurons is a great example. The HuR, HuB, HuC and HuD (ELAVL1-4) proteins preferentially bind to ‘AU’ and ‘U’ rich sequences in 5′ and 3′UTRs of the RNAs. These interactions are key in regulating RNA stability and translation in changing cellular conditions (Lopez de Silanes et al., 2004). In early zebrafish embryos, 3′UTR elements and translational control play crucial roles in regulation and stability of maternal RNAs (Mishima and Tomari, 2016; Winata et al., 2021). Mammalian studies have also identified general motifs via CLIP-seq (cross-linking immunoprecipitation sequencing) that some RBPs bind to, for instance: UGAU, AAUAAA. Interestingly, the IGF2BP3 family of RBPs recognises CWWCATCA and TGCACTAT, to facilitate mRNA transport and enhance stability (Li et al., 2017). In this study, we experimentally validated the YBE motif in *furina* mRNA by *in vitro* RNA structure probing. Although we do not see a perfect recapitulation of the hypothesised 3 bp stem loop structure, we see some support for RNA structure in that region and note that our *in vitro* observations might be confounded by the lack of stabilising trans-acting factors that would normally be present *in vivo*. One possibility is that perhaps there is RNA structure heterogeneity in the region, whereby all these RNAs can generate a structure similar enough to that observed by structure probing, but with *furina* WT having the highest propensity and *furina* SB the lowest propensity for the predicted structure.

We show that Ybx1 interacts with *furina* mRNA at the embryonic stages prior to organ morphogenesis. The phenotype observed in *ybx1* mutant embryos is likely due to a lack of translational control of *furina* mRNA and is consistent with overexpression of FurinA protein. When there is an excess of FurinA protein, cleavage of Spaw is upregulated and the midline of an embryo is saturated with Spaw, which causes bilateral expression and consequently LR asymmetry defects (Tessadori et al., 2015). To date, there are very few studies showing involvement of RBPs in LR asymmetry. One study described translational repression of Bicc1 on *dand5* 5′UTR mRNA and showed a role for this interaction in the establishment of laterality (Maerker et al., 2021). Dand5 is a negative inhibitor of Nodal signalling and probably acts at a different level of the Nodal pathway, downstream of *furina* and Ybx1 regulation. Interestingly, we observed the LR asymmetry defects in approximately 20% of the mutant embryos. Ybx1 is a global translational repressor and it also interacts with the Nodal inhibitors Lefty1 and Lefty2 (Sun et al., 2018; Zaucker et al., 2017). Although in the *ybx1* temperature-sensitive allele the mutation was triggered in late epiboly stage, there is still upregulation of Lefty1 in *ybx1* mutants. During somitogenesis, Lefty1 is expressed at the midline of the embryo preventing Spaw from crossing the midline barrier and ensuring its leftward expression (Lenhart et al., 2011). However, in *ybx1* mutants, upregulation of Lefty1 activity likely represses elevated Spaw. This, as a result, might reduce the severity of the LR asymmetry phenotype in *ybx1^sa42^* embryos.

In addition to the LR organ-positioning defects, we also found that the AV canal in the *ybx1* mutants is enlarged and, consequently, the embryos have retrograde blood flow. This phenotype could also potentially be independent of or in addition to the interaction of FurinA with Spaw. FurinA is a ubiquitously expressed protein that cleaves a range of targets, which are required for correct heart and AV canal development, such as Notch1b in zebrafish (Zhou et al., 2021). Mouse Furin is expressed in a range of cell types and loss of Furin activity in the extra-embryonic and cardiogenic mesoderm leads to yolk sac morphogenesis and heart looping defects (Constam and Robertson, 2000). Moreover, conditional loss of Furin activity in endothelial cells is associated with cardiac valve malformations and ventricular septal defects (Kim et al., 2012). These phenotypes seem similar to the cardiac morphogenesis defects in our *ybx1* mutants with elevated Furin activity, and lend support to the possibility that Furin activity levels are tightly controlled in embryos and any misregulation might lead to defects in heart development. In *Xenopus* and mouse, Furin has been shown to interact with the bone morphogenic pathway (BMP) family proteins, which are also important for heart development (Constam and Robertson, 2000; Cui et al., 1998). The loss of translational control of *furina* in *ybx1* mutants might also disrupt interactions with one of these targets and lead to additional heart defects. It is also possible that Ybx1 might regulate heart morphogenesis through other Nodal-independent mechanisms (Noël et al., 2013).

When we made deletions within the *furina* 3′UTR by CRISPR/Cas mutagenesis using a pair of gRNAs flanking the YBE, we could not recover stable germline mutants. Nonetheless, in somatic mutant embryos in which we disrupted the *furina* X1 3′UTR, we find increased LR patterning defects compared with wild-type or control-injected embryos. Together with the LR patterning, heart morphogenesis and valve defects in *ybx1* mutants, these results support a role for the 3′UTR/Ybx1 regulon in translational control of *furina* and heart development.

There are reports of regulation of the heart development by non-coding molecules, such as microRNAs (miRNAs). Key cardiac development genes such as *tbx2*, *notch1* and *cspg2* are regulated by miRNAs in zebrafish (Kalayinia et al., 2021). Studies in mice have shown that increased levels of miR-25 lead to depressed cardiac function, but inhibition of miR-25 reversed the phenotype and increased survival in a mouse heart failure model (Wahlquist et al., 2014). Studies in humans have shown that reduced amounts of miR-26a, miR-30b and miR-195 in stenotic valves led to increased chance of developing valve leaflet fusion. These miRNAs are thought to be involved in modulation of calcification genes; when their levels are lower, calcium begins to accumulate at the aortic valve leaflet and changes the morphology (Nigam et al., 2010).

We find that *ybx1* mutant embryos have retrograde blood flow at 5 dpf. Retrograde blood flow can be observed normally up to day four in larvae, and is important for modelling of the AV canal and valve development, and mutations in the heart valve precursor gene *klf2a* cause aberrant valve development (Vermot et al., 2009). In *ybx1* mutants, we observe that red blood cells squeeze through the valves back to the atrium. This is similar to a human condition called mitral valve regurgitation, which is characterised by a leaky valve leading to backflow of blood. The extent of the symptoms varies, with some people being asymptomatic and others experiencing dizziness, breathlessness and irregular heartbeat (Adams et al., 2010). The expansion of the atrioventricular canal likely prevents heart valves from shutting properly, and during ventricular contraction the blood cells squeeze between the valves and go back to the atrium of mutant hearts. Therefore, our *ybx1* mutant could serve as a zebrafish model for this disorder and potentially lead to better understanding and future treatments of heart valve regurgitation disorders.

## MATERIALS AND METHODS

### Zebrafish strains

Zebrafish were maintained in accordance with the University of Warwick institutional animal welfare regulations and the UK Home Office guidelines. All fish in this work were maintained in the Tübingen (TU) background. Zebrafish *ybx1^sa42^* fish lines and controls were set up as single pair crosses separated with a divider the previous day; on the next morning, the divider was removed to allow mating. Maternal *ybx1* embryos (M*ybx1*; i.e. progeny of homozygous *ybx1* females mated with wild-type males) as well as maternal zygotic *ybx1* mutants (MZ*ybx1*; progeny of homozygous *ybx1* male and female matings) arrest during early gastrulation regardless of the genotype of the male, whereas paternal *ybx1* (*Pybx1*; progeny of homozygous *ybx1* males mated with wild-type females) develop normally (Kumari et al., 2013). We used both M*ybx1* and MZ*ybx1* embryos in the experiments and they are generally referred to as *ybx1* mutants.

Embryos were kept at 28°C in 0.3× Danieau's solution [17 mM NaCl, 2 mM KCl, 0.12 mM MgSO_4_, 1.8 mM, Ca(NO_3_)_2_, 1.5 mM HEPES, pH 7.6] until 50% epiboly, and afterwards they were shifted to 22°C until the 20-somite stage. Embryos were then shifted back to 28°C until 50 hpf (long-pec stage) for live imaging or fixed in 4% paraformaldehyde, dehydrated and stored in methanol at −20°C. For heart imaging, MZ*ybx1* and wild-type control embryos were collected at the same time and grown at 28.5°C until the 75% epiboly stage. They were subjected to temperature shifts to 22°C until the 21-som stage and shifted back to 28°C until 5 dpf.

### PCR and qPCR amplification

Zebrafish embryos from stages 1 K, 50% epiboly, 10-som, 18-som and adult ovaries were collected and 50 eggs/embryos were used per stage. RNA was initially homogenised in TRIzol reagent (Thermo Fisher Scientific) and then extracted using an RNA miniprep kit (NEB). The integrity of purified RNAs was checked on agarose gel and concentration was measured on the a NanoDrop spectrophotometer. Next, RNAs were reverse transcribed using SuperScript III (Thermo Fisher Scientific). For qRT-PCRs, each cDNA was amplified using *gapdh*, *furina* and *furina* X1 primers in a standard 20 μl PCR reaction [5× GoTaq Green buffer (Promega), dNTPs 10 mM (Promega) of 2 µm each forward and reverse primer, home-made Taq polymerase and sterile water]. For qPCR, we used the 2× Luna Universal qPCR Master Mix (NEB), following the manufacturer's protocol. All primers were tested for efficiency to ensure they were comparable. Normalisation to *18S* rRNA expression was carried out for qRT-PCR. The primer list is available in Table S1.

### *In situ* hybridisation

Embryos were fixed at the required developmental stages overnight at 4°C in 4% paraformaldehyde in PBS and WISH was performed according to established protocols (Thisse and Thisse, 2008).

### Fluorescent RNA synthesis and injections

Plasmids were linearised using NotI restriction enzyme and mRNA was transcribed from purified template DNA using SP6 RNA polymerase. Transcription master mix contained 500 ng of linear purified template, 5× transcription buffer, DTT, ribonuclear capping mix (rATP, rGTP, rCTP; 10 mM each) rUTP (100 mM), ^7^mG(5′)pppG cap (7.5 mM) and Alexa 488-5-UTP (1 mM) or Alexa 546-UTP (1 mM) (Thermo Fisher Scientific) fluorophore. Following transcription samples were treated for 30 min with a Turbo DNase I enzyme and unincorporated nucleotides were removed using mini-spin columns (Bio-Rad). Next, phenol-chloroform purification was performed on the fluorescent RNAs, followed by overnight precipitation in isopropanol and elution in nuclease-free water. RNA integrity was checked on an agarose gel and the concentration and incorporation rate of the fluorophore was checked using a NanoDrop spectrophotometer. Embryos were injected at the 1-cell stage with 10-25 pg aliquots of fluorescent mRNAs, imaged and/or scored at 4-cell stage.

### RNA immunoprecipitation

Zebrafish embryos were collected at following stages: 50% epiboly, 10-som and 18-som, in aliquots of 250 embryos. Embryos were cross-linked in 1% formaldehyde solution for 20 min, rinsed three times with PBS-T (PBS with 0.1% Tween 20) and lysed using RIPA buffer in DEPC water (50 mM Tris-Cl pH 7.5, 1% NP-40, 1% sodium deoxycholate, 0.1% SDS, 1 mM EDTA, 1 M NaCl, 1 M urea, protease inhibitor). Immunoprecipitation was performed according to Kumari et al. (2013) and Zaucker et al.(2017), with an anti-Ybx1 antibody (Sigma-Aldrich, 4F12), followed by qRT-PCR. 5S rRNA expression served as a control for RT-PCR. A list of primers used in RT-PCRs and qPCRs is available in the Table S1.

### Immunofluorescence

Antibody staining was performed as described by Santos et al. (2018). MF20, S46 and ZN-8 antibodies were purchased from Developmental Studies Hybridoma Bank, created by the NICHD of the NIH and maintained at The University of Iowa, Department of Biology, Iowa City, IA 52242, and used according to the manufacturer's recommendations.

### Translation assays and western blot analysis

Full-length *spaw*-GFP and *furina* sfGFP were cloned into pCS2 vector by Gibson assembly and transcribed using the SP6 mMachine kit (Thermo Fisher Scientific). Capped *in vitro*-synthesised RNAs were treated with turbo DNase, phenol-chloroform purified, precipitated overnight in isopropanol and eluted in nuclease-free water. Next, 50 pg of RNA was injected into 1-cell-stage maternal *ybx1^sa42/sa42^* mutant embryos, and 50% of the injected embryos were temperature shifted to 22°C at the 16-cell stage, and 50% of injected embryos were kept at 28°C as controls. At the 512-cell stage, all embryos were collected and lysed in RIPA buffer (50 mM Tris-Cl pH 7.5, 1% NP-40, 0.5% sodium deoxycholate, 0.05% SDS, 1 mM EDTA, 150 mM NaCl) with proteinase inhibitor (Roche). The sample concentration was normalised using a Bradford assay kit (NEB), following which all samples were boiled for 5 min in Laemmli buffer, loaded on a 10% acrylamide gel and western blotting was carried out. Membranes were incubated with anti-GFP HRP conjugated antibody (1:2000; Santa Cruz, sc-9996 HRP) and HRP-conjugated Actin loading control (1:4000; Santa Cruz Biotechnology, sc-47778).

### Confocal imaging

Imaging of immunofluorescence and for reporter translation was carried out using an Andor Revolution Spinning Disk system, with a Yokogawa CSU-X1 spinning disc unit, fitted with a 488 nm laser and captured with an iXon Ultra 888 EMCCD camera. Control, mutant and injected embryos were dechorionated and mounted in a mould (Zaucker et al., 2021) and imaged with identical settings and exposure times. As Ybx1 acts as a general translational repressor in mammalian cells and zebrafish embryos (Evdokimova et al., 2001; Minich et al., 1993; Sun et al., 2018), normalisation of Spaw and Furin translation reporter-injected embryos was carried out with rhodamine dextran injection control as described previously (Zaucker et al., 2017). Images for *z-*stacks were acquired using Andor software and final processing and analysis performed in ImageJ.

### Heart imaging

MZ*ybx1* and wild-type control embryos were collected at the same time and grown at 28.5°C until the 75% epiboly stage. They were subjected to temperature shifts to 22°C until the 21-som stage and shifted back to 28°C until 5 dpf. At 24 hpf, embryos were treated with the phenylthiourea at 0.003% concentration in Danieau's solution to prevent pigment formation. To distinguish between general LR and heart morphogenesis defects, embryos were sorted for heart looping and only embryos with normal looping were assessed for cardiac valve function. Prior to imaging on day 5, embryos were immobilised by adding 25× tricaine solution to Danieau's solution and mounted in 0.6% low melt agarose on a 3-cm glass cover slip-bottom Petri dish for imaging at 13 frames per second using Nikon ECLIPSE Ni microscope with a HAMAMATSU digital camera C11440, ORCA-Flash4.OLT. Time-lapse movies of approximately 30 s to 1 min duration were acquired.

To analyse the direction and velocity of the blood flow in *ybx1* and control embryos, the particle image velocimetry (PIV) application was used in MATLAB (Thielicke and Sonntag, 2021). Each movie was converted in ImageJ using the function ‘find edges’ and uploaded to PIV, the region of interest was set out to be a junction in the AV canal and frames were analysed. Vectors were saved and analysed for direction with a custom MATLAB code.

### RNA structure probing

RNAs were probed *in vitro* as described by Flynn et al. (2016), except that the MgCl_2_ concentration for 3.3× SHAPE reaction buffer was reduced to 3.3 mM (333 mM HEPES, 3.3 mM MgCl_2_, 333 mM NaCl) and probing reactions were conducted at 28°C. Gel images were first quantified by SAFA (Das et al., 2005), and normalised by subtraction (Eqn 1). Upper and lower vigintiles were calculated from the base reactivities for all *furina* transcripts. Finally, reactivities of 1 and 0 were defined by the upper and lower vigintiles for all base reactivities for a given RNA and values below zero were set to zero (Eqn 2):
(1)

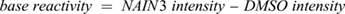


(2)

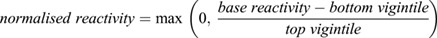


### Generation and analysis of *furina* Δ3′UTR crispants

Several gRNAs targeting the 3′UTR of the *furina* transcript were designed using CHOPCHOP v3 software (Thyme et al., 2016) and CRISPRon (Xiang et al., 2021; Anthon et al., 2022). Off-target analysis was carried out using CRISPRoff to ensure selection of gRNAs with minimal off-target potential, i.e. all gRNAs were selected with high predicted specificity (Alkan et al., 2018; Anthon et al., 2022). The selected gRNAs (Table S1) were synthesised by *in vitro* transcription of PCR product DNA templates with the gRNA sequence fused to a T7 promoter, following an oligo-based strategy (Varshney et al., 2015). Several gRNAs were tested for efficient cutting after co-injection with Cas9 mRNA using a T7 endonuclease (T7E1) assay and two efficient gRNAs spanning an ∼1.3 kb region that contains the YBE and located 60 nt after the stop codon were selected (Table S1). We designed a primer pair that amplifies a 188 bp PCR product from the predicted Δ3′UTR deletion mutant allele, primer F and primer R2. The predicted 188 bp deletion mutant sequence was successfully amplified from gDNA extracted from embryos that had been injected with the two gRNAs with Cas9 mRNA. The PCR product was purified and sequenced to confirm the sequence resulting by fusion of sequences adjacent to the gRNA target sites, with an ∼1.3 kb deletion in the intervening region. Sequences of the gRNAs and genotyping primers are provided in the primer list (Table S1).

For generation of *furin* Δ3′UTR crispants we injected 1-cell-stage embryos with 2 nl aliquots containing 1 μM each of the gRNAs and 1.33 μM Cas9 protein (NEB, M0646T). Injected embryos were kept at 28.5°C until 60% epiboly, incubated at 22°C overnight, and fixed at the 19- to 21-somite stage in 4% paraformaldehyde in PBS. In one experiment, 22 embryos were genotyped using primers F and R2 together with primer R1, which anneals to a sequence that is deleted in Δ3′UTR crispants. Using the three primers together in a single PCR reaction, we can detect wild-type and deletion mutant products in parallel. To rule out non-specific phenotypes arising from toxicity of the injected materials, we used multiple controls: non-injected wild-type embryos, 1 μM Cas9 protein only-injected embryos, and embryos injected with 1 μM Cas9 together with gRNA2 and the control non-cutting gRNAc (instead of gRNA1).

### Statistical analysis

An unpaired, two-tailed Student's *t*-test was used for statistical analysis of heart looping, qPCR datasets and western blot band and fluorescence intensity measurements. Single-factor ANOVA was used to analyse the width of the atrioventricular canal at 3 dpf. Fisher's exact test was used to analyse significance of *spaw* expression in *ybx1^sa42^* mutant embryos and *furina* 3′ UTR crispants and *foxa3* expression. For all statistical analyses, *P*-values are represented as follows: *P<0.05, **P<0.01, ***P<0.001; *P*>0.05 was considered non-significant.

## Supplementary Material

10.1242/develop.201657_sup1 Supplementary information

Table S1. List of primers and guide RNAs
