# Supplementary material for: Translational control of furina by an RNA regulon is important for left-right patterning, heart morphogenesis and cardiac valve function
Source: Development. 2023 Nov 30;150(23):dev201657. doi: 10.1242/dev.201657 (PMC10730018; doi:10.1242/dev.201657)
Supplement: Supplementary information [file develop-150-201657-s1.pdf]

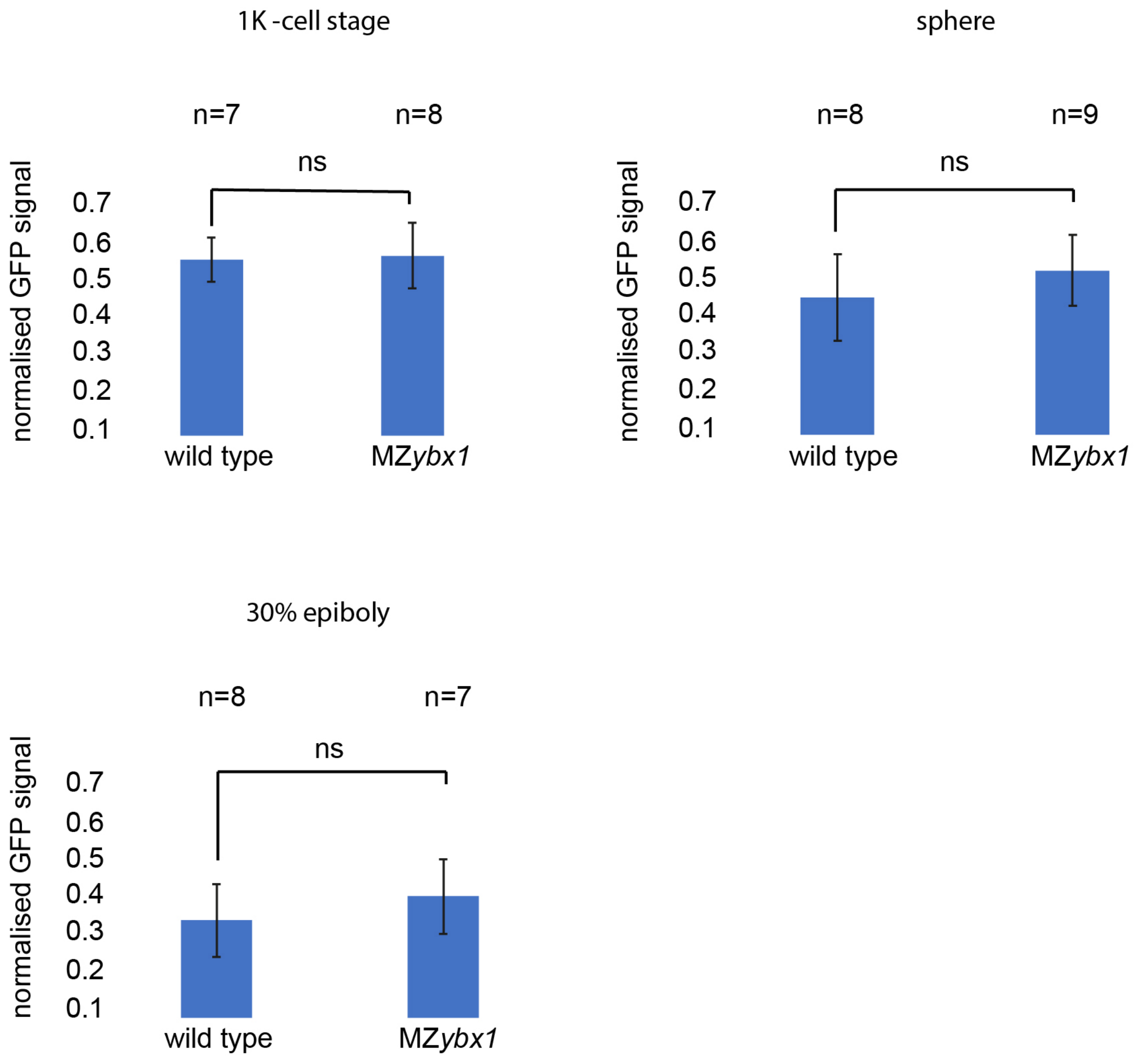

Fig. S1. Total Spaw-GFP fusion protein fluorescence levels are similar in wild type and ybx1 mutant embryos.

Quantification of fluorescence levels across the entire blastoderm at 1K, sphere and 30% epiboly; Spaw-GFP signal was normalised to mCherry mRNA reporter control.

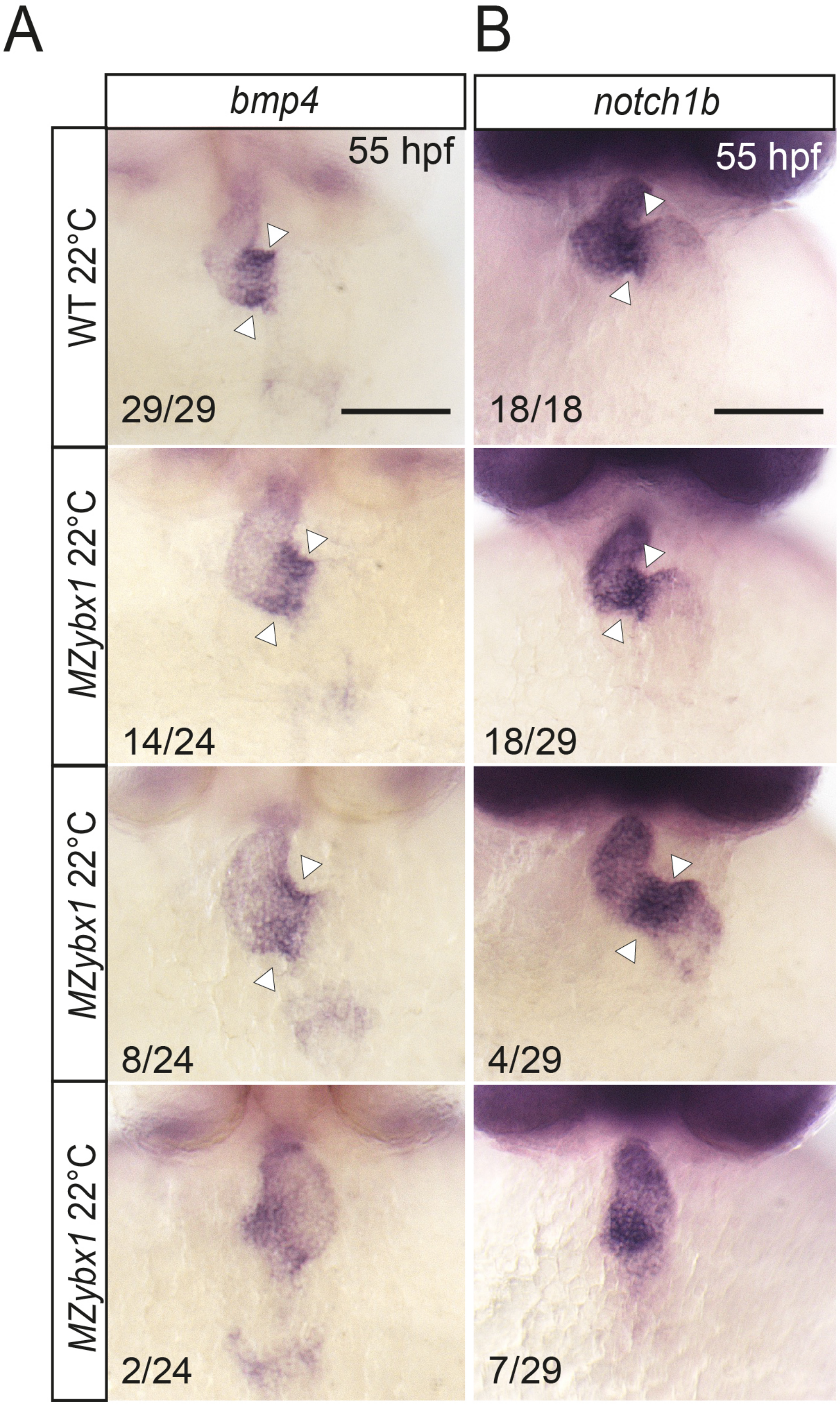

Fig. S2. The heart appears enlarged in MZybx1 mutant embryos at 5 dpf

A) WISH showing bmp4 expression in wild type and MZybx1 mutant embryos. White arrowheads point to the AV canal. Scale bar, 100 µm.

B) WISH with notch1b probe, showing expression in the AV canal at 55 hpf in wild type and MZybx1 mutant embryos. White arrowheads point to the AV canal, scale bar, 100 µm.

Table S1. List of primers and guide RNAs

Available for download at
https://journals.biologists.com/dev/article-lookup/doi/10.1242/dev.201657#supplementary-data

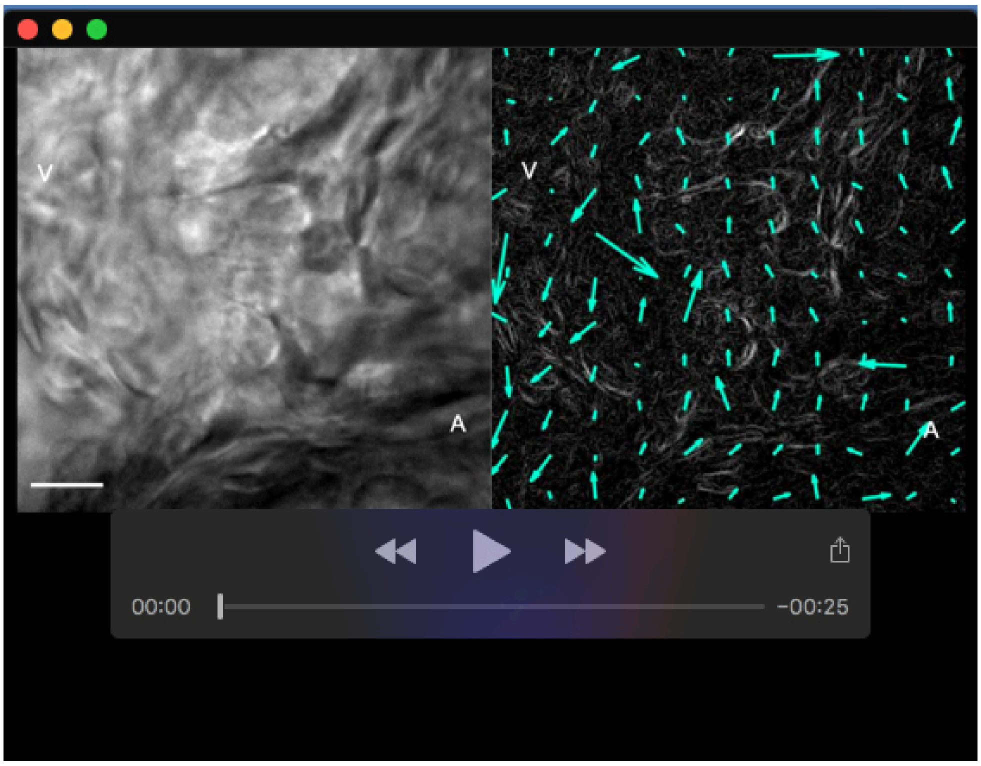

Movie 1. Blood flow is altered in MZybx1 mutants compared to wild type embryos. DIC movie of heart beating in a wild type embryo at 5 dpf (left); PIV analysis (right) shows direction of blood flow, cyan arrows indicate the red blood cells. Images captured at 13 frames per second (fps), V marks ventricle and A marks atrium; Scale bar, 20 µm.

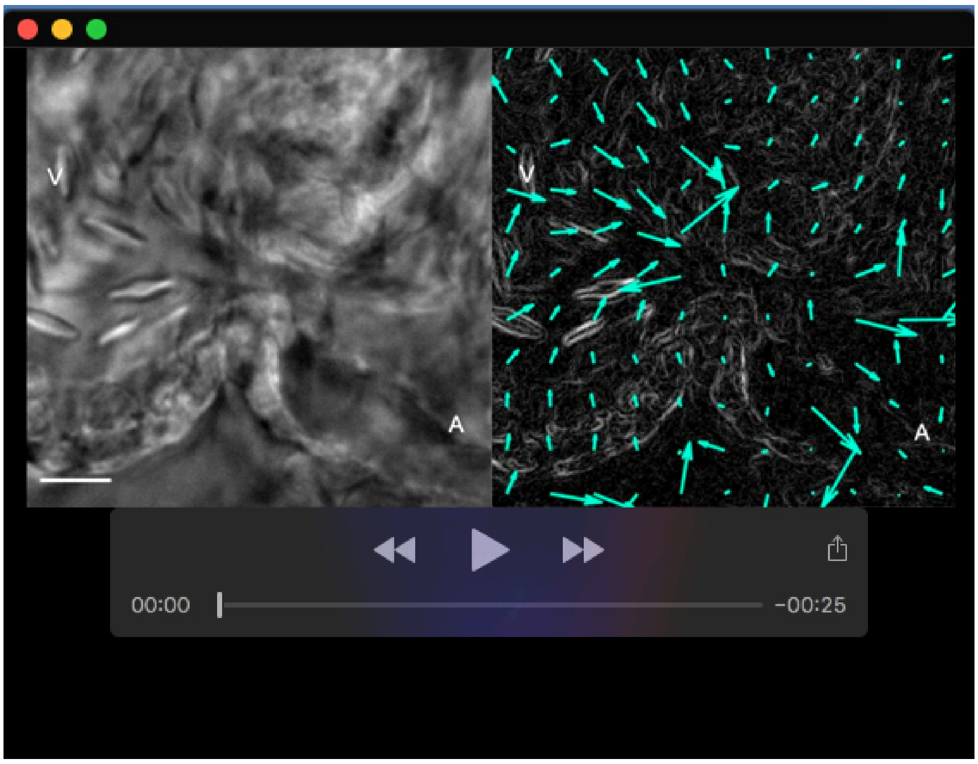

Movie 2. Blood flow is altered in MZybx1 mutants compared to wild type embryos. DIC movie of heart beating in an MZybx1sa42 mutant embryo at 5 dpf showing retrograde blood flow (left). PIV analysis (right) shows the movement of red blood cells. Images were captured at 13 fps; V marks ventricle and A shows atrium; Scale bar, 20 µm
